# Increased Neutrophil-Subset Associated With Severity/Mortality In ARDS And COVID19-ARDS Expresses The Dual Endothelin-1/VEGFsignal-Peptide Receptor (DEspR): An Actionable Therapeutic Target

**DOI:** 10.21203/rs.3.rs-846250/v1

**Published:** 2021-09-13

**Authors:** Victoria L. M. Herrera, Allan J. Walkey, Mai Q. Nguyen, Christopher M. Gromisch, Julie Z. Mosaddhegi, Matthew S. Gromisch, Bakr Jundi, Soeren Lukassen, Saskia Carstensen, Ridiane Denis, Anna C. Belkina, Rebecca M. Baron, Mayra Pinilla-Vera, Meike Muller, W. Taylor Kimberly, Joshua N. Goldstein, Irina Lehmann, Angela R. Shih, Roland Ells, Bruce D. Levy, Nelson Rulz-Opazo

**Affiliations:** Boston University School of Medicine; Boston University School of Medicine; Boston University School of Medicine; Boston University School of Medicine; Boston University School of Medicine; Boston University School of Medicine; Brigham and Women’s Hospital, Harvard Medical School; Berlin Institute of Health and Charité - Universitätsmedizin Berlin, Universität Berlin, Humboldt-Universität zu Berlin; Fraunhofer Institute for Toxicology and Experimental Medicine; Boston University School of Medicine; Boston University School of Medicine; Brigham and Women’s Hospital, Harvard Medical School; Brigham and Women’s Hospital, Harvard Medical School; Fraunhofer Institute for Toxicology and Experimental Medicine; Massachusetts General Hospital, Harvard Medical School; Massachusetts General Hospital, Harvard Medical School; Charité - Universitätsmedizin Berlin, corporate member of Freie Universität Berlin, Humboldt-Universität zu Berlin and Berlin Institute of Health (BIH); Massachusetts General Hospital, Harvard Medical School; Berlin Institute of Health and Charité - Universitätsmedizin Berlin, Universität Berlin, Humboldt-Universität zu Berlin; Brigham and Women’s Hospital, Harvard Medical School; Boston University School of Medicine

**Keywords:** DEspR, neutrophil subset, secondary tissue injury, ARDS, COVID19-ARDS

## Abstract

Neutrophil-mediated secondary tissue injury underlies acute respiratory distress syndrome (ARDS) and progression to multi-organ-failure (MOF) and death, processes linked to severe COVID19. This ‘innocent bystander’ tissue injury arises in dysregulated hyperinflammatory states from neutrophil functions and neutrophil extracellular traps (NETs) intended to kill pathogens, but injure cells instead, causing MOF. Insufficiency of prior therapeutic approaches suggest need to identify dysregulated neutrophil-subset(s) and induce subset-specific apoptosis critical for neutrophil function-shutdown and clearance. We hypothesized that neutrophils expressing the pro-survival dual endothelin-1/signal peptide receptor, DEspR, are apoptosis-resistant just like DEspR+ cancer cells, hence comprise a consequential pathogenic neutrophil-subset in ARDS and COVID19-ARDS. Here, we report correlation of circulating DEspR+CD11b+ activated neutrophils (DESpR+actNs) and NETosing-neutrophils with severity in ARDS and in COVID19-ARDS, increased DEspR+ neutrophils and monocytes in post-mortem ARDS-patient lung sections, and neutrophil DEspR/ET1 receptor/ligand autocrine loops in severe COVID19. Unlike DEspR[−] neutrophils, ARDS patient DEspR+actNs exhibit apoptosis-resistance, which decreased upon *ex vivo* treatment with humanized anti-DEspR-IgG4^S228P^ antibody, hu6g8. *Ex vivo* live-cell imaging of non-human primate DEspR+actNs showed hu6g8 target-engagement, internalization, and induction of apoptosis. Altogether, data differentiate DEspR+actNs as a targetable neutrophil-subset associated with ARDS and COVID19-ARDS severity, and suggest DEspR-inhibition as a potential therapeutic paradigm.

## 1-sentence summary

Circulating DEspR+CD11b+ neutrophils and NETosing neutrophils are associated with severity and mortality in ARDS and COVID19-ARDS.

## Introduction

Acute respiratory distress syndrome (ARDS) and progression to multi-organ failure (MOF) comprise a pathological spectrum of secondary ‘bystander’ tissue injury arising when one’s inflammatory response to an inciting ‘primary injury’ – be it infectious or non-infectious – becomes dysregulated and excessive.^1^ Stopping this feed-forward destructive inflammation in ARDS and MOF remains an important unmet need, as there is no FDA-approved pharmacotherapy able to reduce the high mortality in ARDS from MOF.^2^ The lethality of destructive inflammation is highlighted by the COVID19 pandemic as progression to ARDS and multi-organ failure are accelerated in COVID19, and comprise the major cause of death in severe COVID-19.^3^ Notably, destructive inflammation often exhibits a feed-forward progression to multi-organ failure and death even if the inciting primary injury is resolving, if not finite, such as decreasing bacterial or viral load, one-time sterile trauma. This supports the scientific rationale that effective targeted inhibitors of destructive inflammation can be agnostic of the different primary injury etiologies in ARDS – be it bacterial infection at any site, SARS CoV2, or sterile injury.

As an approach to address the pressing need for novel therapies for ARDS and COVID19-ARDS, we reasoned that comparative analysis and identification of pathogenic commonalities in ARDS and COVID19-ARDS could help identify novel therapies. *A priori,* this pinpoints cytokine storm inhibitors common to both ARDS and COVID10-ARDS. However, the redundancies among cytokine-mediated pathways, and the partial-only reduction in mortality by an IL-6 receptor inhibitor in severe COVID19,^4^ suggest that targeted inhibition of the cellular effectors of the cytokine storm will be required. Among cytokine effector cells, neutrophils have long been implicated in ARDS and progression to multi-organ failure.^1,5^ Activated neutrophils play key roles in multi-organ dysfunction and progression to failure^6^ through neutrophil-mediated microvascular endothelial injury, capillary permeability,^7^ and neutrophil-extracellular trap (NET)-associated endothelial and lung epithelial injury.^8,9^ The central role of neutrophils is supported by the association of increased neutrophil-lymphocyte ratios (NLR) with worse ARDS prognosis^10^ as well as with more severe COVID19 and poor prognosis^11^.

More recently, comparative single cell RNA-sequencing (scRNA-seq) analysis of mild and critically ill COVID19-patients, and non-infected healthy controls, demonstrated molecular evidence supporting the central role of neutrophils,^12^ concordant with other reports^13–15^. However, inhibiting neutrophils effectively to mitigate neutrophil-driven secondary tissue injury safely and effectively has been elusive despite preclinical efficacy in animal models of acute lung injury.^5^ The cumulative low translatability, due most likely to species differences in neutrophil biology and to multifactorial complexities in ARDS pathogenesis not present in preclinical models of acute lung injury, provides scientific basis for *ex vivo* analysis of ARDS patient whole blood samples. The *ex vivo* study of neutrophils within the pathobiological context of other immune cells in the circulation during progression of ARDS towards multi-organ failure or resolution is imperative in the validation of putative therapeutic targets.

Based on insights from the study of DEspR + cancer stem-like cells (CSCs) exhibiting aberrant apoptosis-resistance associated with myeloid cell leukemia (Mcl1) levels, a key apoptosis-evasion protein in cancer,^16^ we reasoned that DEspR + neutrophils would also have survival advantages as Mcl1 levels correlate with neutrophil survival.^17^ Since neutrophil apoptosis is required for efferocytosis, function shutdown and active resolution of inflammation^18^, longer neutrophil survival increases risk for dysregulation and subsequent excessive or hyper-inflammation. Additionally, given that endothelin-1 (ET-1) levels, one of two DEspR ligands,^19^ are elevated in ARDS,^20^ and since ET1 is known to enhance neutrophil activation and functionality,^21^ ET1-mediated DEspR activation could play key pathogenic role(s) in neutrophil-mediated secondary tissue injury in ARDS.

We therefore tested the unifying hypothesis that DEspR + neutrophils comprise an activated neutrophil subset with pathogenic survival advantage over other DESpR[−] activated neutrophils, and whose cumulative increase drives neutrophil-mediated secondary tissue injury in ARDS and COVID19-ARDS leading to multi-organ failure. Here, we studied 1) whether DEspR + neutrophils comprise a neutrophil-subset associated with ARDS clinical severity, mortality, and reported biomarkers of ARDS-severity, as well as, with higher levels of circulating neutrophil extracellular trap formation (NETosis), 2) whether identification of the DEspR + neutrophil subset is reproducible in different research labs and concordant with scRNA-seq findings in severe COVID19, and 3) whether DEspR + neutrophils can be safely inhibited to restore neutrophil apoptosis as a targetable therapeutic neutrophil subset.

## Results

### DEspR + CD11b + human neutrophil subset increased by TLR4 activation

**Figure 1.**
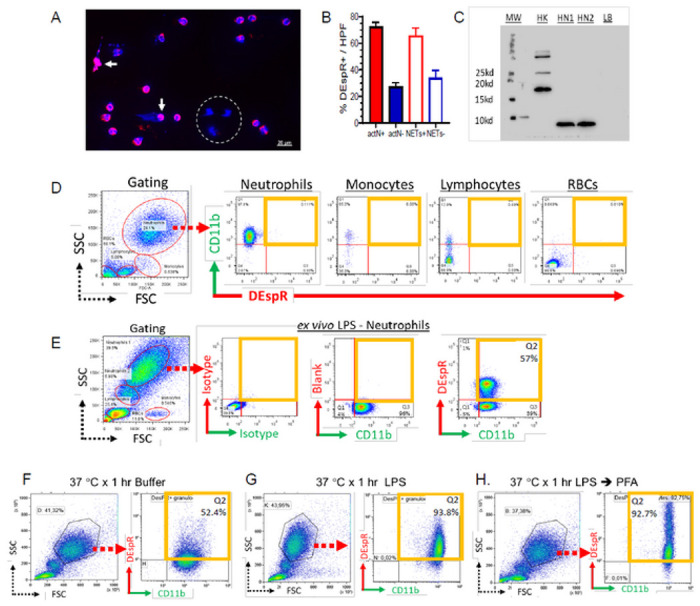
DEspR expression on normal human volunteer (NHV) neutrophils. (**A**) Immunofluorescence of 24-hour old NHV neutrophils: DEspR (hu6g8) ●. DAPI ●. merged ●. DEspR+ NETosing neutrophils(➔). DEspR(−) neutrophils encircled ○. Bar = 20 μm. (**B**) Bar graph showing semi-quantitation of % DEspR+ neutrophils and NETosing neutrophils in 6 high power fields per slide in 3 slides prepared from NHV polled neutrophils. (**C**) Western blot analysis showing DEspR expressed in normal human kidney (HK), neutrophils (HN1) and LPS-stimulated neutrophils (HN2). MW, molecular weight markers. LB, Laemli buffer as negative control. (**D**) Flow cytometry (FCM) analysis of EDTA-anticoagulated NHV whole blood, non-stimulated. Gating of white blood cells for neutrophils, monocytes, lymphocytes and red blood cells (RBC) by their respective clouds determined by differential forward scatter (FSC, x-axis), side scatter (SSC, y-axis), features, and fluorescence markers: anti-CD11b-FITC, anti-DEspR (hu6g8)-AF568. (**E**) FCM analysis of NHV (EDTA-anticoagulated) whole blood stimulated with LPS (75 μg/ml, 1-hr at 37 °C). Panels left-to-right: FSC/SSC gating for neutrophils: isotype controls hIgG4-AF568, mIgG2-AF4S8; FMO-CD11b+): CD11b (X-axis) vs DEspR (Y=axis). (**F-H**) FCM analysis of NHV (Li-heparin anticoagulated) showing increased DEspR+CD11b+neutrophils in 3 different conditions: (**F**) after 1-hr incubation at 37 °C. (**G**) after 1hr LPS-stimulation (75 ng/ml) at 37 °C; and (**H**) after paraformaldehyde permeabilization done after 1 hr LPS-incubation at 37 °C. PFA-permeabilization detects increased DEspR with high (intracellular and cell-surface DEspR) and low (cell-surface) DEspR+ expression.

To determine DEspR-expression in human neutrophils as a potential therapeutic target, we analyzed protein levels by immunofluorescence, western blot and flow cytometry analyses. First, we performed immunofluorescence staining of normal human volunteer (NHV) neutrophils that have survived *ex vivo* for ~ 24-hours. We used a humanized anti-DEspR antibody, cross reactive to human, monkey and rodent DEspR, with a hinge-stabilized [S228P]IgG4 backbone, hu6g8, developed by us and validated for detection of cancer cells expressing DEspR by immunofluorescence, western blot analysis and flow cytometry, and for in vitro and in vivo efficacy in inducing apoptosis in DEspR + tumor cells.^16^ Direct immunofluorescence of 24-hrs old surviving neutrophils detected DEspR expression in multiple compartments: neutrophil nuclei, cell membrane and cytoplasm (Fig. 1–A), consistent with membrane-cytoplasmic-nuclear shuttling observed in cancer cells.^16^ Immunofluorescence staining also detected DEspR + neutrophils with extruded DNA and still intact cell membranes suggesting DEspR+ “vital NETosis”^22,23^, as well as DEspR[−] neutrophils (Fig. 1A). Notably, majority of 24-hour old neutrophils and NETosing neutrophils were DEspR + in these *ex vivo* experimental conditions (Fig. 1B). Next, parallel western blot analysis of whole cell protein isolates detected the expected size DEspR protein in the NHV neutrophils, thus confirming DEspR + expression. Western blot analysis also detected a larger DEspR protein in human kidney (Fig. 1C, Fig. S1A) due to PNGase-sensitive glycosylation, as shown previously in cancer cells.^24^

To further assess DEspR + expression on neutrophils in preparation for ARDS patient studies, we performed flow cytometry (FCM) analysis with double-immunostaining for cell-surface co-expression of DEspR and CD11b. We selected CD11b as a marker of activated neutrophils as CD11b mediates neutrophil-complement system crosstalk, and since CD11b + neutrophils are increased in ARDS patient peripheral blood and in broncho-alveolar fluid.^25^

Flow cytometry analysis of normal human volunteer (NHV) EDTA-anticoagulated whole blood samples detected minimal, if any, DEspR + expression on the cell surface of intact CD11b + activated neutrophils (actNs), monocytes and lymphocytes in baseline conditions, and no expression on red blood cells (RBCs) (Fig. 1D). DEspR expression was increased by standard RBC-lysis step done before antibody binding (Supplementary Fig. S1–B), likely in response to damage associated molecular patterns (DAMPs) released during RBC hemolysis.^26^ Notably, in response to cell stress expected in any *ex vivo* condition, DEspR expression also increased with time from blood sampling > 1-hour be it at 4 °C or at 37 °C (Supplementary Fig. S1–C).

Having validated flow cytometry detection of DEspR + CD11b + neutrophils, we next determined whether toll-like receptor 4 (TLR4)-activation increases DEspR + expression, since TLR4-activation by DAMPs is elevated in both ARDS^27^ and in COVID19-ARDS^28^, and increases CD11b + expression on neutrophils^29^. After incubation of NHV whole blood (EDTA-anticoagulation) *ex vivo* with an established TLR4-activator, bacterial lipopolysaccharide (LPS), flow cytometry detected increased DEspR + expression in majority but not all CD11b + neutrophils (Fig. 1E), identifying a specific DEspR + CD11b + neutrophil subset (DEspR + actNs).

To independently test detection of DEspR + CD11b + neutrophils in NHV blood samples, collaborators performed flow cytometry analysis on Li-heparin anticoagulated NHV whole blood that would allow more robust LPS-activation without EDTA-chelation of Ca^2+^, Mg^+2^ ions. This testing detected higher DEspR + CD11b + activated neutrophils: ~ 52.4% in buffer (Fig. 1F), and ~ 93.5% with LPS challenge (Fig. 1G). To assess potential intracellular stores, collaborators analyzed permeabilized TLR4-activated neutrophils using light fixation (2% paraformaldehyde) only so as not to alter antigenic epitope. Flow cytometry analysis detected higher fluorescence levels of DEspR + expression but same % of DEspR + CD11b + neutrophils (Fig. 1H), compared to non-PFA-permeabilized TLR4-activated neutrophils (Fig. 1G). This observation suggests the presence of intracellular DEspR, in addition to cell surface DEspR, hence higher “total” DEspR + expression. This is supported by membrane, cytoplasmic expression detected by immunocytostaining (Fig. 1A).

Relevant to *ex vivo* analysis, these observations indicate that EDTA-anticoagulated blood exhibit less susceptibility to *ex vivo* experimental changes with increases in time and temperature (Fig. 1E, Fig. S1–C). For quantitative *ex vivo* ARDS patient sample analysis, we therefore used only EDTA anti-coagulated whole blood processed within 1-hour from sampling from hereon, in order to minimize confounders that increase DEspR-expression *ex vivo.* This will avoid overestimating actual circulating levels in patient samples and false positives.

### DEspR + neutrophils and monocytes/macrophages in ARDS patient lung sections

**Figure 2.**
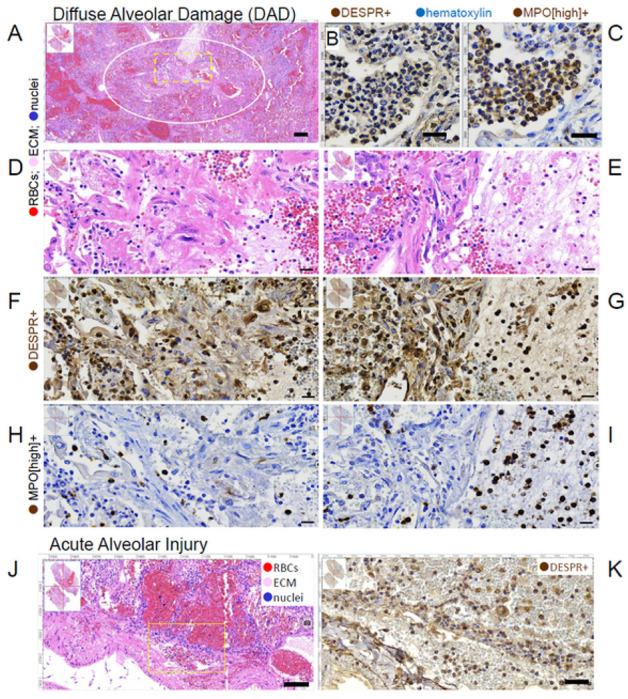
DEspR+ neutrophils and monocytes in ARDS post-mortem lung sections. (**A**). H&E section showing area of diffuse alveolar damage (DAD) in post-mortem lung section (○) from ARDS patient. Yellow dashed □, region of Interest (ROI) analyzed in higher magnification in panels D-I). (**B**) Immunohistochemical-diaminobenzidene (IHC-DAB)-staining of bronchiolar exudate and transmural infiltrates showing DEspR+ expression, and (**C**) myeloperoxidase (MPO)+ expression, hematoxylin as counterstain. (**D**-**E**) Higher magnification of Region of Interest (ROI) #1 (D) and #2 (E.) with diffuse alveolar damage (DAD) shown in (A). (**F**-**G**) IHC-DAB staining of adjacent serial sections showing DEspR+ expression in inflammatory cells in DAD-ROI#1 (F) and ROI#2 (G). (**H**-**I**) MPO+[high-expression] IHC-DAB staining in ROI#1 and #2 show high levels typically seen in neutrophils. The 5X lower levels of MPO+ expression in macrophages were not detected in conditions used: hematoxylin counterstain. (**J**) Representative H&E image of area with acute alveolar injury with infra-alveolar hemorrhages in lung sections also exhibiting diffuse alveolar damage. Yellow box depicts ROI shown in (K). (**K**) Representative IHC-DAB staining for DEspR+ expression in area of acute alveolar injury. DEspR+ neutrophils and monocytes in the intravascular lumen, parenchyma, alveolar walls and intra-alveolar spaces. **B**ar = 20 microns.

To demonstrate pathological basis to study DEspR + immune cells in ARDS patient whole blood samples, immunohistochemistry analyses of post-mortem serial lung sections from patients with ARDS (n = 8) in regions of diffuse alveolar damage (Fig. 2A–2I) as well as, in areas of acute alveolar injury (Fig. 2J–2K) were performed. Using an anti-DEspR mouse-recombinant mAb of hu6g8, hu6g8-m, immunohistochemistry with DAB chromogen (IHC-DAB) was optimized and detected DEspR + neutrophils in intrabronchiolar exudate, along with some DEspR(−) neutrophils with characteristic polylobulated nuclei (Fig. 2B). In adjacent serial sections, IHC-DAB immunostaining conditions limited to detection of only high myeloperoxidase (MPO[high]) + expression typical in neutrophils with 5x higher expression than monocytes^30,31^, detected primarily MPO[high] + neutrophils in the intrabronchiolar exudate (Fig. 2C). In areas of diffuse alveolar damage (DAD) (Fig. 2D, 2E), IHC-DAB analyses of serial sections detected intra-alveolar, intravascular and intraparenchymal DEspR + neutrophils and monocytes (Fig. 2F–G) and MPO[high] + neutrophils (Fig. 2H–I). Similarly, in lung areas of acute alveolar injury present in the same ARDS patient lung section slides (Fig. 2J), immunohistochemistry detected DEspR + neutrophils and monocytes in the intravascular, parenchymal and intra-alveolar spaces (Fig. 2K). These observations validate measuring peripheral DEspR + CD11b + neutrophil- and monocyte-subset levels by flow cytometry as a window into projected pulmonary levels in ARDS or COVID19-ARDS.

### DEspR core-expression network increased in COVID19 neutrophils

To determine pathogenic basis to study DEspR + neutrophils in COVID19-ARDS, we analyzed single cell RNA-sequencing (scRNA-seq) database of healthy control (n = 5), mild (n = 8) and severe (n = 11) COVID19 patient samples. Since DEspR’s ADAR1 RNA-edited transcript^16^ is not discernable in scRNA-sequencing limited to 300 nucleotides from the poly-A sequence of each transcript to ascertain specificity and equivalent representation, we focused on DEspR’s essential expression-network comprised of DEspR’s modulators, ligands, and bioeffect marker represented in the scRNA-seq database of immune cells and epithelial cells in nasopharyngeal and broncho-lavage fluid samples from COVID19 subjects.^12^

**Figure 3.**
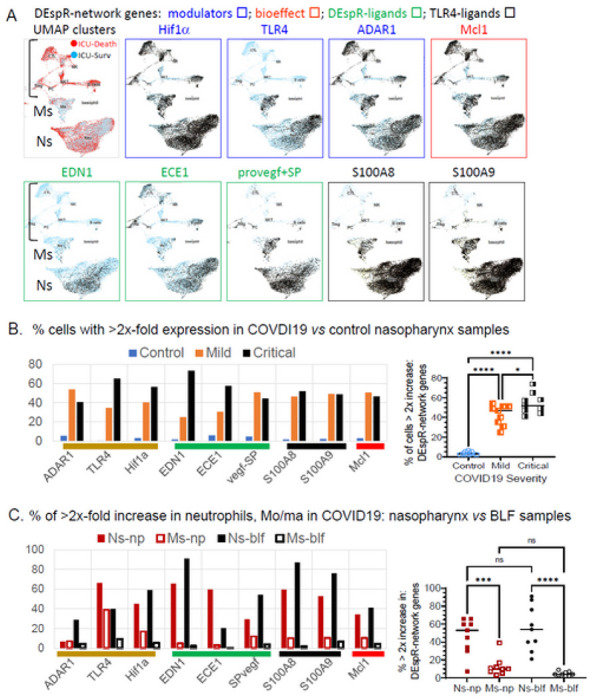
Single cell RNA-seq profiles of genes that modulate DEspR expression: DEspR-expression network. (**A**) Profile of scRNA-seq analysis, showing UMAP cluster depicting neutrophils (Ns). monocytes (Ms) and lymphocytes ([) identified by scRNA-sequence profiles of broncholavage fluid samples from critically ill COVID19 patients (n = 2), and corresponding scRNA-seq levels of DEspR-modulators; HIF1α, modulator of DEspR transcription, at RNA level (Hif1α stabilized in normoxia by activated TLR4), at protein level (ADAR1 RNA-editase), mobilization to cell surface upon activation (TLR4). DEspR-bioefiect marker for pro-survival role (Mcl1). DEspR-ligands endotbelin-1 (EDN1, ECE1), and signal peptide (propeptide VEGF), and TLR4-activators during cell injury – alarmins: S100A8 and S100A9. No expression (●). ≥ 2x expression (●). (**B**) Quantitative scRNA-seq database analysis of DEspR-expression network genes measured as % of cells in sample with ≥ 2x-fold increase in RNA levels in nasopharyngeal samples comparing non-COVID19 control patients (n = 5, mean = sd: 3.1 ± 1.9), mild COVID19 patients (n = 8: 42.6 ± 10.2), and critically ill COVID19 patients (n = 11: 54.1 ± 10.6). Non-parametric one-way ANOVA with Tukey’s multiple comparisons testing; *, p = 0.0226. ****, p <0.0001. (**C**) Quantitative scRNA-seq database analysis of DEspR-expression network genes comparing % ≥ 2x expression for each gene among neutrophils (Ns) and monocytes-macrophages (Ms) in nasopharyngeal (np) and broncho lavage fluid (blf) samples, (n = 9 genes in DEspR-exp Network per cell type) in COVID19-patient samples. Average % expression of all 9 genes in Ns-np; (mean ± sd; 46.78% ± 19.9); Ms-np: (12.7% ± 10.7); Ns-blf; (55.3% ± 25.1), and Ms-blf (4.2% ± 2.8). Non-parametric one-way ANOVA with Sidak’s multiple comparisons testing: ***. p = 0.0006; ****, p < 0.0001.

Comparative scRNA-seq analysis showed that positive modulators of DEspR-transcription (TLR4 and Hif1-α), DEspR RNA-editing for translation (ADAR-1 RNA-editase), DEspR cell-surface mobilization (TLR4), TLR4-endogenous activators (alarmins S100A8/S100A9) and bioeffect prosurvival marker (Mcl1) are predominantly expressed in neutrophils, along with DEspR’s two ligands, endothelin-1 (ET1 or EDN1), and the signal peptide in VEGFA-propeptide (spVEGF) (Fig. 3A). Interestingly, neutrophil expression of the endothelin converting enzyme (ECE1), required for release of ET1 from its pro-peptide (Fig. 3A) suggests neutrophil self-sufficiency to produce mature ET1, and hence, a putative ET1/DEspR autocrine loop. In contrast, transcripts for other ET1 receptors: ETA-receptor (ETAR) and ETB-receptor (ETBR), VEGF-A receptors: (KDR, Flt-1), and other alarmins receptor: (AGER) were minimally, if not, detected in neutrophil scRNA-sequences (Supplementary Fig. S2A).

Notably, expression levels of all 9 genes representing DEspR’s expression and functional network are significantly increased in COVID19 compared to healthy controls (Fig. 3B), and in neutrophils compared to monocytes-macrophages in nasopharyngeal and broncho-lavage COVID19 samples (Fig. 3C). The basis to study neutrophils is further supported by scRNA-seq documentation of increased expression of receptors to cytokines increased in ARDS^32^ and/or in COVID19-ARDS^33^, such as: IL-6, IL-8, IL-1β, IL-18, and TNF-α^34^ (Supplementary Fig. S2G). These observations support the role of neutrophils as effectors of the “cytokine storm” leading to destructive inflammation manifesting as ARDS and/or multi-organ failure, be it COVID19^3^ or bacterial pneumonia.^32^

### Increased DEspR + CD11b + neutrophil-subset In ARDS and COVID19-ARDS

**Figure 4:**
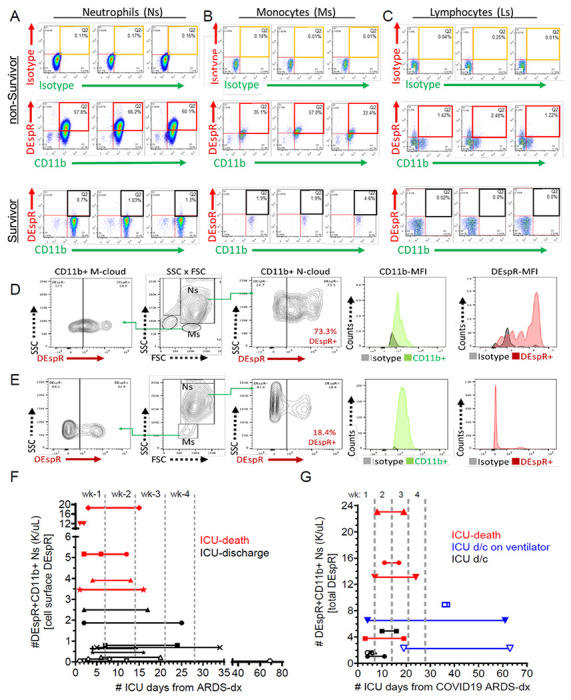
Analysis of DEspR+ neutrophils in ARDS and COVID-19-ARDS patients. (A-C) Representative FCM analysis, done in triplicates, of neutrophils (Ns), monocytes (Ms) and lymphocytes (Ls) in non-survivor vs survivor patient with ARDS. Corresponding isotype controls vs double immunotyping with anti-DEspR (hu6g8) and anti-CD11b. Quadrant 2 (Q2) for DEspR+CD11b+ neutrophils, monocytes and/or lymphocytes. (D-E) Representative FCM-analysis of PFA-fixed samples from patient with COVID-19-ARDS, mechanically ventilated, 61 days in the ICU (D) compared to (E) COVID19-ARDS patient discharged after 6 days in the ICU. CD11b+DEspR+ neutrophils (Ns) (contour plot and histogram of fluorescence intensities), and monocytes (Ms). (F) Graph of duration of ICU-stay (days) from day of FCM-analysis of DEspR+CD11b+Ns (1st symbol) until discharge or death (2nd symbol), stratified by level of number (#) of cell surface DEspR+CD11b+ neutrophils (K/μL) detected. Time zero marks day of ARDS diagnosis in non-COVID19 ARDS, and in (G) COVID19-ARDS. d/c, discharge; wk, week. No FCM-analysis on day of discharge or death.

To study the potential clinical relevance of the DEspR + neutrophil-subset, we performed a prospective pilot observational study of consented patients diagnosed with ARDS based on the Berlin Definition, regardless of acute disease etiologies (sepsis, pneumonia, cardiac arrest, surgery) and comorbidities (Supplementary Table S1 for demographics). First, we ascertained DEspR-specific immunotyping of whole blood samples from ARDS patients by validating our gating strategy for flow cytometry (Supplementary Fig. S3A–E), DEspR-specific detection in duplicates (Supplementary Fig. S3F–S3I) and reproducibility in triplicates (Fig. 4A, Supplementary Fig. S3J). With this ascertainment, we prospectively studied 19 ARDS patients first, then 11 COVID19-ARDS patients in the ICU at Boston Medical Center.

Flow cytometry (FCM) analysis comparing extremes in the clinical spectrum across subjects in the pilot study, a non-survivor with ARDS-multi-organ failure (MOF) compared with ARDS survivor discharged from the ICU in 4 days showed increased cell surface expression of DEspR on CD11b + activated neutrophils (Fig. 4A) and monocytes (Fig. 4B), and on CD11b[−] lymphocytes (Fig. 4C) in the ARDS-nonSurvivor in contrast to minimal DEspR + expression in CD11b + neutrophils, monocytes, and lymphocytes in the ARDS-survivor (Fig. 4A–4C). Fluorescence intensity histograms corroborate DEspR-specific immunotyping and differential expression in triplicates (Supplementary Fig. S3J). With experimental ascertainment of DEspR-specific immunotyping, from hereon, studies were done with duplicates.

Similarly, we also observed differential DEspR + expression in COVID19-ARDS patients in the ICU requiring mechanical ventilatory support (Supplementary Table S1 for demographics). For safety reasons, we studied disinfected (4% paraformaldehyde or PFA) whole blood samples from COVID19-ARDS patients, and performed FCM analysis within 1 hour from blood draw. FCM analysis representing extremes of the clinical severity spectrum also revealed increased total #DEspR + N-counts and monocytes (M)-counts in a patient with severe COVID19-ARDS requiring 61 days intensive care unit (ICU)-care (Fig. 4D), compared with a patient with milder COVID19-ARDS discharged after 6 days in the ICU (Fig. 4E).

Observing marked differential levels at the polar ends of the clinical spectrum of severity, we next stratified mortality outcomes in ARDS (Fig. 4F) and COVID19-ARDS (Fig. 4G) patients by respective levels of DEspR + CD11b + neutrophil-counts (thousand K/μL whole blood). Per level of DEspR + N-counts, we graphed the length of stay in the ICU until discharge or death, and observed a parallel trend of increased DEspR + N-counts with mortality in the graphs. Aside from corroborating the identification of the DEspR + CD11b + neutrophil subset, these observations provide scientific basis for quantitative analysis of emerging differential modulation between survivors and non-survivors in ARDS and COVID19-ARDS.

### Association of DEspR + CD11b + neutrophils with ARDS severity and mortality

**Figure 5:**
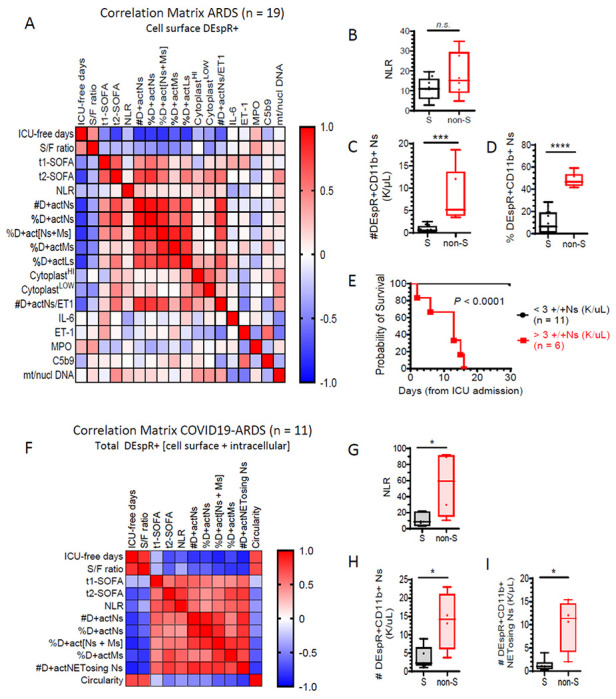
Correlation matrix analysis of DEspR+ neutrophils, clinical parameters and plasma biomarkers in ARDS and COVID19-ARDS. (A) Correlation matrix ARDS: Spearman Rank Correlation, n = 19 patients with ARDS diagnosis (Berlin definition) of cell surface DEspR+ expression levels (% or number #) in CD11b+ activated neutrophils (actNs), monocytes (actMs), and lymphocytes (actLs) and corresponding correlation with 1] clinical parameters of severity [ICU-free days at 28-days, defined as (28 - # of ICU-days) with ICU-death = −1, and > 28-days in the ICU = 0; PsO2/FiO2 or S/F ratio, Sequential Organ Failure Assessment (SOFA) scores on day of FCM-analysis (t1-SOFA), SOFA on day of ICU-discharge or ICU-death (t2-SOFA)]; 2] plasma biomarkers reported to be elevated in ARDS by others [interleukin-6 (IL-6), endothelin-1 (ET1), myeloperoxidase (MPO), terminal complex of complement (sC5b9), and mitochondrial/nuclear DNA ratio (mt/nucl DNA), and 3] NETosis parameters: DEspR+ cytoplast levels with high or low granularity (SSC) on flow cytometry. See Table 1 for specific values: rho, P-values with power > 0.8. (B) ARDS: neutrophil-to-lymphocyte ratio (NLR) between survivors (S) n = 12, (mean ± sd: 10.96 ± 5.4) and non-survivors (non-S) n = 6 (18.03 ± 11.21), Mann Whitney p = 0.33 (n.s). (C) ARDS: number (#) of cell-surface DEspR+CD11b+ neutrophils (Ns) detected in ARDS-survivors: n = 12, (mean ± sd: 0.8035 ± 0.8) vs non-survivors: n = 6 (8.1 ± 6.0); two-tailed Mann Whitney p = 0.0001 (***), effect size Hedges’ g with 4% correction: 2.03). (D) ARDS: Comparison of % of DEspR+CD11b+ neutrophils in ARDS: survivors: n = 13, (10.3 ± 10.0) vs non-survivors: n = 6, (48.2 ± 6.3); two-tailed Mann Whitney p < 0.0001 (****), effect size Hedges’ g with 4% correction: 4.03. (E) ARDS: Kaplan-Meir Survival curve analysis with threshold for DEspR+CD11b+ neutrophil-counts set at 3 K/μL whole blood as determined from Fig. 5C. Log rank (Mantel-Cox) test Chi square 20.56, P < 0.0001, Hazard Ratio (Mantel-Haenszel) 90.5, 95% CI of ratio: 12.91 to 634.7. (F) Correlation matrix COVID19-ARDS: n = 11 (severe COVID19 requiring mechanical ventilation). Parameters as defined in Fig. 5A, with addition of # of DEspR+CD11b+ NETosing neutrophils (D+actNETosing Ns), and circularity index of neutrophils (< 0.8 indicative of NETosing neutrophil, see Methods). (G) COVID19-ARDS: comparison of neutrophil-to-lymphocyte ratio (NLR) between survivors: n = 7 (mean ± sd: 10.7 ± 7.7) and non-survivors: n = 4 (55.3 ± 41.4). two-tailed Mann Whitney p = 0.0242 (*), effect size Hedge’s g with 4% correction: 1.73. (H) COVID19-ARDS: comparison of DEspR+CD11b+ neutrophil-counts (K/μL) in whole blood between survivors: n = 7 (mean ± sd: 3.8 ± 2.99) and non-Survivors: n = 4 (13.8 ± 7.9), two-tailed Mann Whitney p = 0.04 (*), effect size Hedge’s g with 4% correction: 1.82. (I) COVID19-ARDS: #DEspR+ NETosing neutrophil-counts (K/μL) whole blood, survivors: n = 7 (mean ± sd: 1.3 ± 1.29) and non-Survivors: n = 4 (10.0 ± 5.7). Two-tailed Mann Whitney p = 0.0121 (*), effect size Hedge’s g with 4% correction: 2.4.

To assess clinical relevance of the DEspR + neutrophil subset, we performed correlation matrix analysis on a panel of DEspR-based markers, clinical markers of ARDS severity, and plasma biomarkers associated with neutrophil-mediated secondary tissue injury, and endothelin-1, one of two DEspR ligands (Fig. 5A, Table 1). To assess clinical severity, we studied ICU-free days at day 28 from ARDS diagnosis, ARDS severity (PsO2/FiO2 or S/F ratio), and Sequential Organ Failure Assessment (SOFA) scores on the day of sampling for flow cytometry analysis analysis (t1-SOFA) and on day before ICU-discharge or death (t2-SOFA). We also studied several biomarkers pertinent to ARDS pathogenic events: IL-6 (cytokine storm), soluble C5b9 (terminal complex of complement activation), myeloperoxidase or MPO (neutrophil activation), plasma mitochondrial to nuclear DNA ratio (vital NETosis-released mitochondrial DNA normalized to cell-free DNA) and DEspR + CD11b + cytoplasts (anuclear remnants of suicidal NETosis).

In ARDS, Spearman rank correlation matrix analysis detected strong negative correlation – correlation coefficient (r_S_ or *rho*) > 0.6, P value < 0.05, power ≥ 0.8 – between the absolute number of DEspR + CD11b + activated neutrophils and ICU free days at day 28 (Fig. 5A, Table 1). Concordantly, other DEspR-based parameters, such as % of DEspR + CD11b + neutrophils or monocytes or lymphocytes, also showed significant negative correlation with ICU-free days at day28 (Table 1). Interestingly, DEspR + CD11b + neutrophil-counts (#) and %DEspR + CD11b + neutrophils correlated strongly with SOFA scores at discharge from the ICU or prior to ICU-death (t2-SOFA) (Table 1), suggestive of potential prognostic correlation depicted in Fig. 4F. In contrast, neutrophil-lymphocyte ratio (NLR), IL-6, MPO, nor sC5b9 levels did not correlate with ICU-free days or with t2-SOFA score (Fig. 5, Table 1), thus differentiating DEspR + expression-based markers.

These observations are supported by analysis of difference in means between ARDS-patient survivors and non-survivors showing no significant difference for NLR (Fig. 5B), but significant differences (Mann Whitney p = 0.0001) with large effect size (Hegde’s g > 0.8) for DEspR + CD11b + neutrophil-counts (#) (Fig. 5C) and % DEspR + CD11b + neutrophils (Fig. 5D). Kaplan Meier survival curve analysis with a threshold for DEspR + CD11b + neutrophil-counts set at 3,000/μL whole blood showed significant differences in survival (P < 0.0001) (Fig. 5E).

Similarly, in COVID19-ARDS pilot group, Spearman rank correlation matrix analysis also showed significant, strong, negative correlation of DEspR + CD11b + neutrophil-counts with ICU-free days at day 28 from ARDS diagnosis, and with ARDS severity S/F ratio (Fig. 5F, Table 2). Interestingly, the sum of %DEspR+[monocytes and neutrophils] correlated with ICU-free days at day 28 with higher Spearman *rho* correlation coefficient, significance and power than either alone (Table 2). This observation suggests DEspR + CD11b+[neutrophil-monocyte] intravascular-interactions likely involved in systemic tissue injury in ARDS progression to multi-organ failure, as observed in acute glomerular injury.^35^

Notably, the neutrophil lymphocyte ratio (NLR) showed significant albeit less robust correlation with ICU-free days at day 28 in COVID19-ARDs. Comparative analysis of COVID19-ARDS survivors and non-survivors showed significant differences in means with large effect size for both NLR (Fig. 5G) and DEspR + CD11b + neutrophil-counts (Fig. 5H). A retrospective analysis of COVID19-ARDS patients requiring ventilatory support at BMC corroborate significant differences in NLR (Fig. S4A–S4D), concordant with reports that increased NLR is an independent preidcotr of mortality in ARDS and COVID19.^36^ In contrast, C-reactive protein did not show correlation (Supplementary Fig. S4E to S4H).

### Association of DEspR + NETosing neutrophils with mortality and severity in COVID19-ARDS

**Figure 6.**
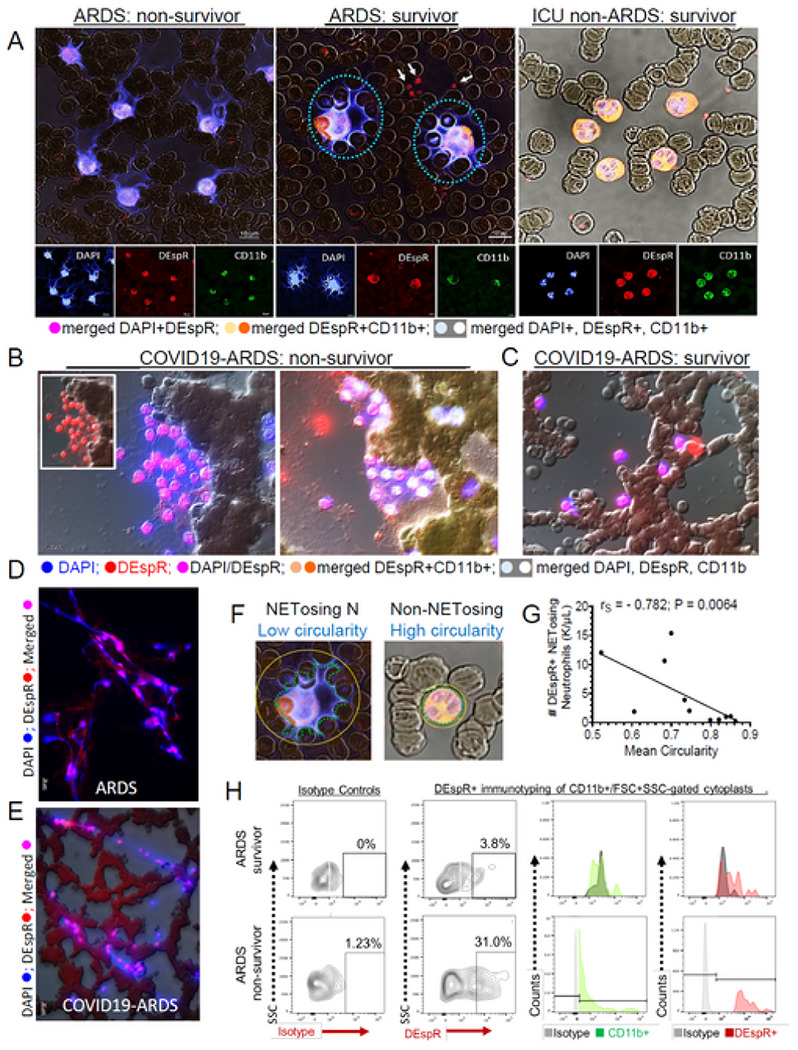
DEspR+ NETosis signatures in patients with ARDS and COVID19-ARDS. (**A**) Representative epifluorescence/DIC images of IF-stained blood smears from ARDS-non-survivor* ARDS-survivor, and non-ARDS critically ill patient in the ICU, DEspR+ NETosing neutrophils (dashed ○) and cytoplasts (➔). anuclear (DAPI−) red blood cells (RBCs) relief images in background. Bar = 20 μm. (**B**) Representative epifluorescence/DIC images of IF-stained blood smears from COVID19-ARDS non-survivor and survivor. IF-staining identical to panel A: inset: DEspR+ NETosing neutrophils prior to merged DAPI+DEspR+ image of NETosing neutrophils with extruded DNA-interconnections among neutrophils. Middle panel: triple-merged DEspR+CD11b+DAPI+ NETosing neutrophils with extruded DNA-interconnections. (**C**) Representative COVIDl9-ARDS-survivor showing minimal NETosing neutrophils. (**D-E**) Representative image of filamentous DEspR-bound extruded DNA networks in IF-stained blood smears from ARDS-patient (**D**) and COVID19-ARDS patient (**E**). (**F**) Analysis of circularity to distinguish NETosing neutrophil with low circularity from non-NETosing neutrophils with circularity index approaching 1.0. (**G**) Spearman rank correlation analysis of # DEspR+CD11b+ neutrophils and mean circularity index (average from n > 500 neutrophils per patient) in COVID19-ARDS patients (n = 11): negative correlation (r_s_ = −0.782, P = 0.0064). (**H**) Flow cytometry analysis of FSC SSC and CD11b+ gated cytoplasts comparing levels in ARDS survivor (3.8%) vs ARDS non-survivor (29.77 %).

To assess formation of neutrophil extracellular traps (NETs) increasingly implicated in severe COVID19,^37,38^ we performed immunofluorescence staining to visualize and quantify NETosing neutrophils in whole blood cytology slides prepared from COVID19-ARDS patients. Using high-resolution confocal imaging of immunofluorescent-stained DEspR + CD11b + neutrophils in patient blood smears prepared within 1 hour from blood draw, we detected differential levels of DEspR + CD11b + NETosing neutrophils in ARDS non-survivor, compared with ARDS-survivor and ICU-patient non-ARDS survivor (Fig. 6A). Similarly, we detected DEspR + CD11b + NETosing neutrophils in COVID19-ARDS non-survivor (Fig. 6B) in contrast to COVID19-ARDS survivor (Fig. 6C). Additionally, we detected interconnecting networks of NETosing neutrophils in COVID19-ARDS non-survivors (Fig. 6B), and DNA-strand networks with DEspR + subcellular ‘beads’ attached to the DNA in both ARDS (Fig. 6D) and COVID19-ARDS patient cytology samples (Fig. 6E).

In order to quantify DEspR + CD11b + NETosing neutrophils (Ns), we used shape analysis (Fig. 6F). Semi-quantitative confocal microscopy in collaboration with Nikon Bioimaging Laboratory distinguished NETosing neutrophils with low circularity index, from non-NETosing neutrophils with expected high circularity (see Supplementary Methods). Quantitative analyses of COVID19-ARDS patient samples spanning hundreds of neutrophils per slide showed significant strong negative correlation of #DEspR + NETosing neutrophils with mean circularity index per patient (Spearman *rho* = 0.78, p = 0.006, power > 0.8) (Fig. 6G). With this correlation, we used a circularity index < 0.8 to identify NETosing neutrophils for quantitative analyses.

In COVID19-ARDS patients, the number (#) of DEspR + CD11b + NETosing neutrophils correlated strongly with three clinical measures: 1] outcome at day-28 (ICU-free days at day-28), 2] degree of hypoxemia (SpO2/FiO2 or S/F ratio), and 3] severity of multi-organ failure (SOFA score at end of ICU-stay) (Fig. 5F, Table 2). Significant differences in means between survivors and non-survivors was also detected (Fig. 5I). This contrasts D-dimer levels obtained during ICU which did not exhibit significant difference in means be it peak levels or average levels while patients were in the ICU (Supplementary Fig. S4–I to S4–L). Notably, scRNA-seq profile for PADI4 linked to suicidal NETosis is minimally expressed in neutrophils with only 1.4% of neutrophils expressing PADI4 > 2X fold (Fig. S2E).

Having detected DEspR + CD11b + cytoplasts with NETosing neutrophils on immunostained whole blood cytology slides (Fig. 6A, middle panel), we analyzed levels of cytoplasts in the circulation by flow cytometry, since cytoplasts are released during suicidal NETosis.^39^ Flow cytometry analysis detected elevated DEspR + CD11b + cytoplast levels in ARDS subjects (Fig. 5A, Table 2) and in COVID19-ARDS (Fig. 6H), however, no significant correlations were observed (Fig. 5A, Table 2). Elevated DEspR + cytoplasts and DEspR + CD11b + neutrophils were detected in an independent pilot study of patients with sepsis, and sepsis-ARDS in contrast to none in healthy donors (Supplementary Fig. S5A–S5D) using a different methodology wherein whole blood samples were enriched for white blood cells via inertial microfluidic separation from RBCs^40^ (Supplementary Fig. S5D–S5F).

### Ex vivo DEspR-inhibition induces apoptosis in ARDS-patient and NHP neutrophils

**Figure 7.**
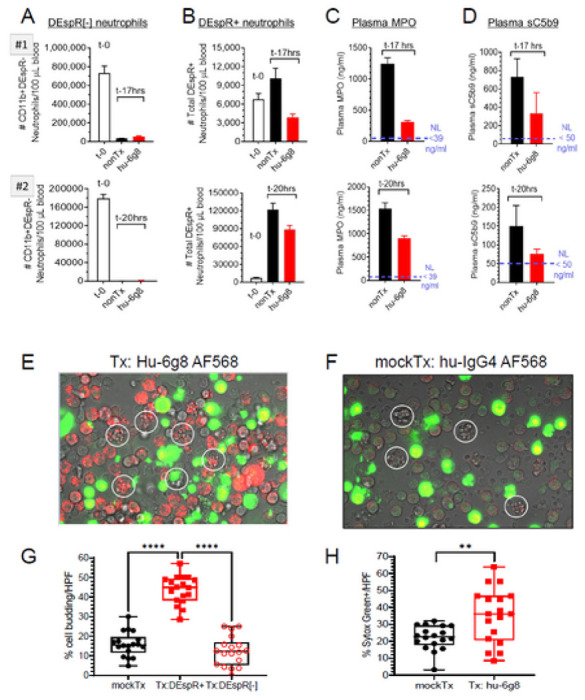
Effects of DEspR-inhibition on neutrophil survival: ex vivo analysis of ARDS-patient and NHP neutrophils. (**A-D**) Analysis of DEspR[−] and DEspR+ neutrophils obtained from patients with ARDS at baseline (< 1 hr from sampling), and after incubation with/without hu6g8 treatment (100 μg/ml) at 37 °C with simulated shear for 17 hrs (patient #1), or 20 hrs (patient#2). Flow cytometry assessed number of (**A**) DEspR[−] vs (**B**) DEspR+ surviving neutrophils compared to baseline. ELISA analyses of (**C**) MPO and (**D**) soluble terminal complex of complement (sC5b9) after *ex vivo* hu6g8 treatment. Normal MPO and sC5b9 levels notated. (**E**) Representative image at t-12hrs from video-recorded live cell imaging of NHP-white blood cells (WBCs) documented to have >90% DEspR+CD11b+ neutrophils among all neutrophils, and exposed to 10 μg/ml hu6g8 AF568 antibody at 4 °C x 30 minutes to eliminate non-specific cell uptake by macropinocytosis or endocytosis. DEspR+ (●) NHP-neutrophils, apoptotic cell budding (encircled ○), and Sytox Green (SytoxG)-positive membrane permeable (●●). (**F**) Representative t-12 hr image of isotype hu-IgG4 AF568 shows minimal to no isotype-AF568 (●) uptake: constitutive apoptosis cell budding (encircled ○). SytoxGreen-positive staining in cells with loss of cell membrane integrity (●●). (**G**) Quantitation of cells exhibiting apoptotic-typical cell budding per high power field (n = 18 HPFs/group, 15-56 cells/HPF), % mean, 95% CI of means: mockTx (16%, 13-19%), TxDEspR+ (44%, 40-47%), TxDEspR[−] (12%, 8.6-16%). One way ANOVA with Tukey’s multiple comparisons test: p < 0.0001 (****). (**H**) Quantitation of SytoxG+ non-viable cells per high power field (n = 18 HPFs/group, > 16-50 cells/HPF), mean, 95% CI of mean for mockTx isotype control (22.2, 18.6-25.7), for hu6g8Tx (35.2, 27.3-43.2): two tailed t-test p = 0.0033 (**).

To determine targetability and bioeffects of DEspR-inhibition, we analyzed bioeffects of *ex vivo* treatment of ARDS patient whole blood with humanized anti-DEspR IgG4^S228P^ antibody, hu6g8, for 17–20 hours overnight with rotation to prevent aggregation. Controls comprised of patient-specific mock-treated control and baseline control (Fig. 7A). Comparative FCM-analysis showed that compared to baseline levels and after 17–20 hrs *ex vivo* incubation at 37 °C, DEspR + neutrophils increased in number indicating longer survival or delayed apoptosis compared with minimal number of DEspR[−] neutrophils (Fig. 7A–7B). In contrast, after DEspR-inhibition via hu6g8-treatment, ARDS patient samples showed decreased number of DEspR + neutrophils, as well as lower myeloperoxidase (MPO) (Fig. 7C) and soluble terminal complex of complement (sC5b9) (Fig. 7D) plasma levels compared to > 2-fold higher levels in respective mock-treated controls. These observations indicate induction of neutrophil apoptosis and function-shutdown of neutrophil-complement system reciprocal co-activation after 17–20 hours of DEspR-inhibition via hu6g8-treatment. Importantly, neutrophil scRNA-seq profile for CD47 is minimal, with only 0.3% - 0.91 % of neutrophils with > 2x fold CD47 (n = 19 COVID19 patients) (Fig. S2F).

To further test that DEspR-inhibition induces apoptosis in DEspR + CD11b + neutrophils, we performed live cell imaging of non-human primate (NHP) neutrophils exposed to fluorescently labeled hu6g8-AF568 or fluorescently labeled human IgG4-AF568 isotype control for 20 minutes at 4°C to avoid non-specific endocytosis. We selected NHPs as model system as NHP-to-human neutrophils are more similar than human-to-mouse neutrophils.^41^ With this set-up, we first show suitability of Rhesus macaque NHPs as model for study by presence of circulating DEspR + CD11b + neutrophils detected via flow cytometry using identical conditions to ex vivo analysis of ARDS patient samples (Supplementary Fig. S6A–H).

Next, we tested for target engagement, internalization, and induction of characteristic apoptosis cell-budding bioeffects by confocal live cell imaging of NHP neutrophils. We first exposed NHP neutrophils to either AF568-labeled anti-DEspR hu6g8 antibody (treatment) or IgG4-isotype (mock-treatment) control for 20 minutes at 4 °C to avoid non-specific endocytosis. After removing excess unbound antibody, 24-hour live cell imaging was initiated with video-recordings. At t-45 minutes, live-cell images detected target engagement and internalization of hu6g8-AF568 antibody (Supplementary Fig. S6–I) but not in the isotype control. Specificities were confirmed throughout with representative t-12 hr timepoint images (Fig. 7E, Fig. 7F). Live cell imaging showed more apoptotic cell budding changes in NHP-neutrophils with internalized hu6g8. In the isotype control, apoptotic cell budding was detected concordant with neutrophil constitutive apoptosis. SytoxGreen impermeable dye uptake marked loss of cell viability. Both cell death indicators increased with time.

At the 12-hour midpoint, quantitation of apoptotic cell changes and SytoxGreen-positive non-viablity were done. Representative 12-hr live cell images show hu6g8-target engagement, internalization and apoptotic cell budding in DEspR + neutrophils (Fig. 7E) compared to minimal uptake of isotype-AF568 control by NHP neutrophils (Fig. 7F). Quantitative analysis of 18 high power fields (HPFs) with 20–50 cells/HPF representing three independent experimental fields of view showed that hu6g8 induced apoptosis in DEspR + neutrophils significantly greater than levels seen in isotype-treated control NHP cells (Fig. 7G). Importantly, hu6g8 induced apoptosis greater than constitutive apoptosis occurring in DEspR[−] cells unaffected by hu6g8 treatment (Fig. 7H). Interestingly, loss-of-viability staining by Sytox Green occurred in neutrophils not undergoing apoptotic cell budding, and was also slightly greater in hu6g8-treated neutrophils compared with isotype mock-Tx controls (Fig. 7H), indicating that DEspR-inhibition may facilitate other programmed cell-death in neutrophils via decreased CIAP2 as observed in anti-DEspR mAb-treated pancreatic cancer stem cells.^42^

## Discussion

Data showing the correlation of DEspR + CD11b + activated neutrophil levels with severity and mortality in both ARDS and COVID19-ARDS delineate a pathogenically relevant neutrophil-subset, DEspR+ “rogue” neutrophil-subset, that exhibits longer survival than DEspR[−] neutrophils and a predisposition to NETosis in circulation. Notably, these neutrophil phenotypes – delayed apoptosis and pro-NETosis – are concordant with previous observations of pathogenic neutrophil phenotypes in ARDS patients.^1–43^

The subset-specific expression pattern of DEspR and its co-expression with CD11b but not in all CD11b + activated neutrophils, are concordant with constraints arising from the need for concurrent expression of DEspR’s multiple modulators: Hif1α and TLR4 for DEspR transcription, ADAR1 for RNA-editing of the DEspR transcript for translation, and activated TLR4 for mobilization to the cell surface. Among the modulators, expression of ADAR1 could be the ‘gate-keeper’ of DEspR + neutrophil subsets as ADAR1 is detected in 29.2% of neutrophils, in contrast to higher levels of neutrophil expression for Hif1a (54%) in critically ill COVID19 broncho-lavage fluid and nasopharyngeal sample neutrophils. This observation is supported by increased ADAR1 localized to neutrophils in ARDS patient lung Sect. ^44^

Presence in pulmonary vascular lumen and in lung areas with diffuse alveolar damage (DAD) and acute alveolar injury, pathological hallmarks in ARDS, validate informativeness of flow cytometry analysis of DEspR + CD11b + neutrophil-subset levels in whole blood samples. Notably, the cell-surface mobilization of DEspR upon TLR4-activation ties the DEspR + neutrophil subset with neutrophil TLR4-activation upon docking of SARS-CoV2 spike protein with neutrophil TLR4 at higher affinity *in silico* than with the spike protein receptor, ACE2^45^. This provides a pathogenic mechanism for direct, hence early activation of TLR4 + neutrophils without need for neutrophilic infection as observed.^12^

Additionally, direct TLR4-activation and CD11b + induction in neutrophils by serum S100A8/A9 alarmins, the prototype DAMPS^46^ found to be elevated in ARDS^47^ and COVID19-ARDS^48^, further ties the DEspR + CD11b + neutrophil-subset to ARDS and COVID19-ARDS. Functionally, as alarmins and TLR4-activation provide a self-sustaining neutrophil activation loop, activated TLR4-induced DEspR upregulation provides a mechanism for delayed apoptosis. This combination of functionalities provides a putative mechanism for feed-forward progression of neutrophil-mediated secondary tissue injury as seen in severe ARDS and COVID19-ARDS, and would be concordant with the observed association of increased DEspR + CD11b + neutrophils with severity and mortality in ARDS and COVID19-ARDS.

The detection by direct visualization of neutrophils with characteristic extruded DNA and retained cell membrane^49^ in ARDS and COVID19-ARDS patient whole blood cytology slides, and detection of elevated mitochondrial to nuclear DNA ratio in plasma, together indicate neutrophils undergoing vital NETosis^50^ in the circulation with extrusion of mitochondrial DNA^51^. The identification of DEspR + expression on said NETosing neutrophils, and significant correlation of #DEspR+/CD11b + vital NETosing neutrophils with clinical measures of severity in COVID19-ARDS, altogether implicate vital NETosis in pathogenesis of progressive multi-organ failure. The detection of NETosis derivatives on the same immuno-stained cytology slides with NETosing neutrophils, such as long DNA-strands with DEspR+ ‘beads’ and DEspR + CD11b + cytoplasts, suggest a dynamic continuum of NETosis paradigms in the circulation in ARDS and COVID19-ARDS.

Intuitively, the observed DNA-strand and interconnections among NETosing neutrophils with their extruded-but still attached DNA fragments could be projected to contribute intravascular biophysical impedance to vascular flow and concomitant low-flow ischemia, thus predisposing to multiorgan dysfunction, as well as microvascular occlusion with or without micro-thromboses. These observations provide insight into why low-flow or micro-ischemic events in different organs persist despite pharmacological thromboprophylaxis or anti-thrombotic treatment.^52^ Additionally, the microvascular flow impedance from DNA-strand and/or from vital-NETosing neutrophil interconnections provide pathogenic concordance with reported severe hypoxemia despite high lung compliance deduced to be due to ventilation/circulation flow mismatch.^53^ More importantly, the detection of DEspR + expression on vital NETosing neutrophils provides an actionable therapeutic target to pre-empt NETosing neutrophils in the circulation.

Data showing that DEspR-inhibition leads to apoptosis in ARDS patient samples and NHP samples supports DEspR as an actionable therapeutic target to induce apoptosis in the dysregulated, apoptosis-resistant neutrophil-subset^54^ implicated in progressive secondary tissue injury leading to ARDS and/or multi-organ failure^55^. Coupled with strong correlation with multiple clinical severity measures, targeted-inhibition of DEspR with endpoint induction of neutrophils apoptosis comprises a much-needed therapeutic paradigm with potential advantages. First, data showing that anti-DEspR hu6g8 induced neutrophil apoptosis and prevented terminal complex of complement sC5b9 increase, suggests function-shutdown of neutrophil-complement system reciprocal-interactions,^56^ and possibly also NETs-induced complement activation.^57^ Second, since neutrophil apoptosis is required for neutrophil function shutdown, clearance and neutrophil-initiation of resolution,^58^ restoration of neutrophil constitutive apoptosis upon DEspR-inhibition could then be expected to promote active resolution of dysregulated hyperinflammation.

While more studies are needed to elucidate mechanisms, the emerging mode-of-action of DEspR inhibition presents a valid pathway to meet therapeutic goals required to stop neutrophil-mediated tissue injury^1^ by inducing neutrophil apoptosis for function shutdown. Additionally, with 99.1% of neutrophils in a representative COVID19 scRNA-profile not expressing CD47 “don’t eat me signal,” induction of neutrophil apoptosis by anti-DEspR antibody can be expected to proceed to efferocytosis. Similarly, with 98.6% of neutrophils not expressing PADI4, anti-DEspR therapy can play a pivotal role in decreasing NETosis-mediated pathogenesis in ARDS and COVID19-ARDS. Third, non-inhibition of DESpR[−] CD11b + activated neutrophil subsets to fight infections and initiate active resolution mechanisms elucidates an inherent safety profile.

Lastly, consideration for potential side effects, especially in the context of acute kidney injury as part of multi-organ failure in ARDS, highlights known DEspR + expression in human medullary tubular epithelial cells. In the presence of immunoglobinuria, anti-DEspR antibody passing through the glomerulus could present potential on-target tubular epithelial effects, but unlikely as antibody functionality will be altered in the increasingly acidic and hyperosmotic milieu in the kidney medullary lumen. Altogether, data identify the DEspR + CD11b + neutrophil subset as an actionable therapeutic target whose targeted inhibition has the potential to slow progression of multi-organ failure in ARDS and COVID19-ARDS, with minimal, if any, projected side effects. These data and insights provide foundational basis for further study.

### LIMITATIONS:

We acknowledge the limitations of prospective pilot observational studies with n = 19 ARDS and n = 11 COVID19-ARDS, and n = 19 COVID19 scRNA profiles. However, pilot observations of flow cytometry data provide per patient informativeness into an actionable therapeutic target in real time, as well as key insights into clinical application feasibility in the critical care setting. With a focus on the study of neutrophils, we did not evaluate other cells with cytotoxic capabilities. We acknowledge the inherent limitations of the study of COVID-ARDS whole blood samples treated with 4% PFA to inactivate SARS CoV2 virus [final 2% PFA], hence parallel independent studies are forthcoming in other medical centers. However, PFA-fixed COVID19 patient samples also provide key insight into differential intracellular stores of DEspR, concordant with modulation of DEspR via its essential expression network in response to pathogenic events in ARDS and COVID19-ARDS.

## Methods

### Study design

Different tasks in this interdisciplinary pilot observational study among different collaborators were compartmentalized in order to attain blinding of researchers during task-performance. The following tasks were compartmentalized: a] patient screening, b] consenting and blood sampling, c] processing of blood for flow cytometry and FlowJo analysis, d] clinical data collection, e] laboratory testing – ELISAs, etc; f] cytology slides preparation from whole blood; g] immune-fluorescence staining, h] confocal microscopy imaging and semi-quantitative measures; i] analysis of collated laboratory and clinical data. The diagnosis of ARDS was determined in real time by review of ICU diagnoses, and checked by clinician collaborators post-hoc blinded to all experimental tasks, such as flow cytometry, cytology and ELISA results.

### Study subjects

All subjects were identified in the ICU under study protocols approved by the Institutional Review Board (IRB) of Boston University (IRB H-36744). Each subject’s legal authorized representative gave written informed consent for study participation in compliance with the Declaration of Helsinki.

We enrolled 19 ARDS patients in the pre-COVID19 pandemic period, and 11 COVID-19 ARDS patients admitted to the intensive care unit (ICU) at Boston Medical Center. ARDS diagnosis was based on clinical diagnosis using the Berlin Definition. COVID-19 ARDS patients were ascertained as COVID-19 positive by SARS-CoV-2 PCR testing. Additional data were obtained prospectively from 16 COVID-19 ARDS patients to examine the time-course during ICU-hospitalization and correlation of other known markers with survival: neutrophil lymphocyte ratio, C-Reactive Protein and D-Dimer. Collaborators enrolled NHVs (MM), patients with severe COVID19 in the ICU for bronchial-lavage fluid studies (RE), healthy donors and patients with sepsis-ARDS for inertial microfluidic separation (BDL, RMB, MPV) according to respective institutional guidelines.

### Blood collection

Whole blood (3 or 6 mls) was collected via pre-existing indwelling peripheral vascular lines into K2-EDTA vacutainer tubes (FisherScientific, MA) from patients hospitalized in the ICU at Boston Medical Center by the ICU-nurse. COVID-19 patient EDTA-anticoagulated blood samples were immediately fixed with one volume of 4% PFA. Both Non-COVID and COVID-19 blood samples were processed for flow cytometry analysis within 1 hour from blood collection. Platelet poor plasma was isolated and frozen at −80°C for future testing within 2 hrs from blood draw. Cytology slides were done within 1 hour from blood draw.

### Flow cytometry analysis of blood samples [See Supplementary Methods for details.]

At BUSM, EDTA-anticoagulated blood samples from non-COVID ARDS subjects (100 μL per tube, x 2–3 replicates) were processed for flow cytometry within 1-hour from blood sampling. [See Supplementary Methods for detail] Flow cytometry buffer comprised of Hank’s balanced salt solution plus 2% heat-inactivated FBS as blocking agent; staining antibodies: 10 μg/ml of AF-647 labeled hu6g8 mAb, or the corresponding human IgG4-AF647 isotype IgG4, and 2.5 μg/ml anti-CD11b-AF488 or the corresponding mouse IgG1 kappa isotype control, AF-488; staining done at 4 °C x 30 minutes with rotation and protected from light; after staining, cells were fixed in 2% PBS-buffered PFA pH 7.4 at 4 °C, followed by RBC lysis at RT. After final wash, stained cells were resuspended in 400 μl HBSS 2% FBS, filtered and analyzed on a BD LSR-II flow cytometer. Analysis was done using FloJo Flow Cytometry Analysis Software (www.FloJo.com). Controls used were: both fluorescence minus one (FMO) controls, both isotype controls, compensation beads for both staining antibodies to check labeled antibody quality.

For disinfected COVID19 blood samples (2%PFA-fixed), samples were washed 3 times with 8 volumes of HBSS + 2% FBS to remove residual fixative prior to processing for flow cytometry as described above. Each test sample run in duplicates.

At BWH, EDTA-anticoagulated whole blood samples were processed 2–3 hours from sampling and white blood cells were separated from RBCs via Inertial Microfluidic Separation validated previously for neutrophil characterization.^40^ Flow cytometry was performed immunotyping for CD45, CD66b and DEspR at room temperature x 20 min, and analyzed on an LSR-Forteza. [See Supplementary Methods for Details.]

### Western blot analysis

Western blot analysis was done essentially as described^42^ using equal amounts of total cellular protein extract (25 μg) isolated from human neutrophils. Neutrophil cell extracts were prepared by cell homogenization in 3 volumes of 1 x Laemmli buffer (Bio-Rad). Human kidney protein extract was used as control. Proteins were size-separated on a 15% Tris-HCL SDS-PAGE (Bio-Rad) and transferred to PVDF membrane (Bio-Rad). The Western blot was reacted with anti-DEspR antibody (hu6g8) at 20 μg/ml for 18 hours at 4 °C with shaking. Immunoreactive proteins were detected by chemiluminescence using the ECL Western Detection kit (Thermo Scientific 34077).

### Immunohistochemistry of tissue sections

We analyzed postmortem human lung sections from patients with clinical diagnosis of ARDS, and pathological diagnosis of diffuse alveolar damage (DAD). Immunohistochemistry was performed at Horus Scientific, Inc using DAB (3,3′-diaminobenzidine) and hematoxylin counter stain. Chimeric anti-DEspR hu6g8 with mouse IgG2a backbone was used at 1:100 dilution (~ 10 μg/ml), and anti-human myeloperoxidase antibody 1:50 dilution. Primary antibodies were incubated for 16 hours at 4 °C. Negative controls were run without primary antibodies, positive controls were run using DEspR + xenograft tumor Sect. ^42^

### scRNA-Seq database analysis

scRNA-Seq data of two patients with critical COVID-19 disease courses (WHO stage 4), covering nasopharynx, protected specimen brush swabs of the airways, and bronchial lavage fluid were obtained from the UCSC Cell Browser generated by studies performed at Charité - Universitätsmedizin Berlin and Berlin Institute of Health. Patient cells were processed using the 10X Chromium system with v3.1 chemistry. Primary analysis was performed using Cell Ranger 3.2.0 with a hg19 reference genome, followed by removal of ambient RNA using SoupX 1.2.2. Preprocessing and primary of analysis of the scRNA-Seq data were performed using Seurat 3.1.4. For details on patient characteristics, sample processing, and data analysis, please refer to Chua et al.^12^ Visualization of the expression of genes of interest was performed using the UCSC Cell browser and confirmed using Seurat 3.2.2 of original datasets. Expression values shown are normalized to the total count of unique molecular identifiers (UMIs) per cell.

### Ex-vivo LPS treatment of human normal volunteer (HNV) neutrophils

[See Supplementary Methods for details.]

At Fraunhofer ITEM, heparinized whole blood was stored on ice until processing and used within 1-hour after collection. Whole blood (100 μl) samples were washed with 1 ml of ice cold assay buffer, and cells were incubated in 100 μl of assay buffer containing bacterial endotoxin lipopolysaccharide LPS (100 ng/ml; Escherichia coli serotype 0111:B4) or assay buffer as control for 1 h at 37°C. The reaction was then stopped, cells washed, then resuspended and cells were stained with hu6g8-PE (10 μg/ml) and CD11b-FITC for 30 min on ice under constant stirring in the dark. Cells were washed to remove unbound antibodies, fixed for 10 min at 4°C, followed by RBC lysis. The cell pellet was resuspended in 250 μl flow cytometry buffer and was analyzed within 2 hours using a Beckman Coulter Navios 3L 10C flow cytometer and data analyzed using Beckman Coulter Kaluza 2.1 Software.

At BUSM, 100μl EDTA-anticoagulated whole blood samples (n = 6) were exposed to 75–100 μg/ml LPS at 37 °C x 1-hour, then subjected to FCM analysis as described above.

### Plasma level analysis of biomarkers by ELISA and Quantitation of Mitochondrial DNA

Individual ELISA protocols were performed as per manufacturer’s instructions with the following sample dilutions: For MPO ELISA (abcam cat# ab195212) plasma dilution 1:1000; for C5b-9 ELISA (MyBioSource cat# MBS2021557) plasma dilution 1:100; for IL-6 ELISA (Abcam cat# ab46027) plasma dilution 1:2; for ET-1 ELISA (abcam cat# ab133030) plasma dilution 1:2.

To compare the levels of mitochondrial to nuclear DNA in human plasma samples we used the NovaQUANT™ Human Mitochondrial to Nuclear DNA Ratio Kit (SIGMA-Aldrich cat# 72620-1KIT) as per manufacturer’s instructions. The kit measures the mtDNA copy number to that of nuclear DNA by Real-Time PCR of specific mitochondrial and nuclear genes. Plasma DNA was isolated from 200 uL of plasma using the Quick-cfDNA Serum & Plasma Kit (Zymo Research, cat# D4076) as per manufacturer’s instruction.

### Immunofluorescence staining of NETosing neutrophils

Cytology slides were prepared by capillary action from EDTA anticoagulated whole blood (10 μL) samples on a Superfrost Plus Microscope slide (Fisher Scientific, cat# 12-550-15) within 1-hour from blood sampling. Cytology smears were air dried for 10 minutes then fixed with 100 % Methanol (chilled to −20 C) for 10 min. Fixed slides were stored dry in −20°C freezer for future immunostaining.

Immunofluorescence (IF)-staining to detect NETosing neutrophils was done as described previously.^59^

### Fixed Cell Imaging of Blood Smears (NETosing quantification)

Immunofluorescence imaging was performed as contract research service at Nikon Imaging Laboratory (Cambridge MA). Slides were imaged with a Nikon Ti2-E Widefield microscope equipped with a Plan Apo λ 20x objective and Spectra LED light source and controlled by NIS-Elements. Briefly, an automated, JOBS routine in NIS-Elements was used to image 100 evenly spaced positions along an entire slide. At each position, focus was automatically adjusted with the Perfect Focus System (PFS) and then sequential images with the 395 (Blue), 470 (Green) and 555 (Red) nm LED light sources to detect DAPI (nuclei), Alexa Fluor 488 (CD11b) and Alexa Fluor 568 (DEspR, hu6g8), respectively. Each stack of 100 images was then processed with a General Analysis 3 algorithm in NIS-Elements to segment the nuclei, measure their circularity (Circularity = 4π [area/perimeter^2^], area of minimum circle enclosing NETosing neutrophil, perimeter of NETosing neutrophil with all DNA-extrusions], and quantify the signal intensity of any co-localized CD11b and DEspR expression. Data were exported to a CSV file where the final scoring is completed in Excel. [See Supplementary Methods for details.]

### Ex-vivo anti-DEspR treatment of ARDS patient blood samples

One ml of freshly obtained blood samples were incubated overnight at 37 °C with or without anti-DEspR mAb (hu6g8 at 100 μg/ml). After incubation half of the samples were subjected to FACS analysis as described above and the other half was processed for plasma isolation. Plasma MPO and C5b-9 levels were determined with corresponding ELISA kits as described above.

### Quantitation of apoptotic cell changes and viability after anti-DEspR treatment of non-human primate (NHP) DEspR + CD11b + neutrophils by live cell imaging

See Supplementary Methods for details.

Briefly, whole blood from Rhesus macaque NHP provided by Biomere (Biomere Biomedical Research Models, Inc., Worcester MA) was analyzed by flow cytometry to determine the number of DEspR + CD11b + activated neutrophils. White blood cells (WBCs) were then obtained, washed and resuspended in Hank’s Balanced Salt Solution (HBSS) + 2% Fetal Bovine Serum (FBS). WBCs were counted, divided into aliquots and incubated with 10 μg/ml Alexa Fluor 568-conjugated hu6g8 antibody or Alexa Fluor 568-conjugated IgG4 isotype antibody for 20 minutes at 4°C. Cells were washed to remove unbound antibody, then concentrated at to approximately 10^8^ cells/mL, then loaded into imaging device. Live cell imaging was performed using a microfluidic chip with three parallel conjoined microfluidic channels, and a confocal microscope (Ti2-E microscope equipped with Nikon A1R HD25 point scanner and 60X Plan Apo λ Oil objective) housed within a temperature and CO_2_-controlled incubator. Images were then acquired every minute for the first 9 hours, and then every 5 minutes for 15 hours thereafter, for a total of 24 hours observation time. At 15 minutes into imaging, Sytox Green (Thermo-Fisher) was added into the imaging media for each chip at a final concentration of 1:6000.

### Statistical analysis

For demographics, statistical comparisons of clinical parameters between the non-COVID and COVID-19 ARDS subjects we used the Fisher Exact test (GraphPad Prism v9.0.1) comparing corresponding proportions, except for age, S/F ratio and SOFA score which were done by using a two-tailed Mann Whitney (GraphPad Prism v9.0.1). For survivor vs non-survivor group comparisons, we used the two-tailed Mann Whitney test (GraphPad Prism v9.0.1) with effect size calculated via Hedge’s g with 4% correction. Correlations were calculated by using the Spearman Rank Order correlation test (GraphPad Prism v9.0.1) and power calculations determined by using SigmaPlot 11.0 software. All data sets conformed to the assumptions of each specific statistical test. P < 0.05 was considered statistically significant, sufficient power 0.8.

## Supplementary Material

Supplement 1

## Figures and Tables

**Table 1.** Spearman correlation results for non-COVID ARDS cohort.

	Clinical measures of severity							
	ICU-free days		S/F ratio		t1-SOFA		t2-SOFA	
Biomarkers	r_S_	P-value	r_S_	P-value	r_S_	P-value	r_S_	P-value
**CBC-differential**								
NLR	−0.2675	0.2964	−0.2399	0.3681	−0.1222	0.6382	0.0764	0.7776
**Flow cytometry analyses**								
**#D+actNs**	***−0.8000***	***0.0002***	−0.3753	0.1520	0.4185	0.0954	***0.7057***	***0.0031***
**%D+actNs**	***−0.7768***	***0.0001***	−0.4062	0.1190	0.5572	0.0163	***0.7897***	***0.0003***
**%D+act[Ns+Ms]**	***−0.8055***	***0.0003***	−0.5147	0.0517	0.4392	0.0899	0.5366	0.0420
**%D+actMs**	***−0.6438***	***0.0088***	−0.4754	0.0749	0.1232	0.6474	0.1935	0.4879
**%D+actLs**	*−0.7899*	*0.0003*	−0.3591	0.1714	0.2383	0.3544	0.5244	0.0392
**Cytoplast** ^**HI**^	−0.2911	0.2540	0.0144	0.9598	−0.2428	0.3441	0.2652	0.3165
**Cytoplast^Low^**	−0.3929	0.1195	−0.4448	0.0978	0.1319	0.6103	0.4719	0.0666
**ELISA – plasma levels**								
IL-6	0.0232	0.9273	−0.0486	0.8587	0.3484	0.1565	−0.0050	0.9864
ET-1	−0.0682	0.8011	0.0769	0.7966	0.3546	0.1938	0.0277	0.9232
MPO	0.2840	0.2533	0.3932	0.1319	−0.2310	0.3564	−0.2521	0.3252
sC5b9	−0.2904	0.2425	0.1016	0.7061	0.0765	0.7629	0.0578	0.8239
**PCR analysis**								
mt/nucl DNA	−0.0359	0.8978	0.1473	0.6158	−0.0647	0.8104	0.4435	0.0990

n = 19 subjects ARDS, all cause [pre-COVID19 pandemic].

#, total number K/mL whole blood; %, % from total Ns, Ms, or Ls; actNs, CD11b+ activated neutrophils; cytoplast^HI^, high granularity (SSC) cytoplast; cytoplast^LOW^, low granularity cytoplast; D+, cell surface DEspR+ expression; ET-1, endothelin-1 plasma levels; IL-6, interleukin-6 plasma levels; Ls, lymphocytes; Ms, monocytes; MPO, plasma myeloperoxidase levels; mt/nucl DNA, mitochondrial to nuclear DNA ratio; Ns, neutrophils. NLR, neutrophil to lymphocyte ratio; sC5b9, plasma levels of soluble complement terminal C5b9-complex.

**Clinical measures of severity:** ICU-free days at day 28 = [28 – number of ICU days] with NonSurvivors = −1 and Survivors > 28 ICU days = 0. S/F ratio, SpO2/FiO2 ratio as a measure of hypoxemia severity. t1-SOFA, sequential organ failure assessment (SOFA) score on day of flow cytometry analysis; t2-SOFA, SOFA score at end of ICU stay (day before ICU-discharge or ICU-death).

**Statistical analysis:** Spearman Rank Order Correlation coefficient *rho* (r_S_) effect size: strong r_s_ 0.6 – 0.79; very strong r_s_ 0.8 – 1.0 monotonic relationship between paired data. Data points used are peak values for subjects with multiple FCM analyses. Spearman Correlation Coefficient r_S_ > 0.605, alpha < 0.05, Power > 0.8 (*red italics*).

**Table 2.** Spearman Rank Correlation matrix analysis: COVID19-ARDS subjects requiring ventilator support.

	Clinical Measures of ARDS severity							
Biomarkers:	ICU-free days		S/F ratio		t1-SOFA		t2-SOFA	
CBC-differential	r_S_	P-value	r_S_	P-value	r_S_	P-value	r_S_	P-value
Neutrophil-lymphocyte ratio	***−0.6201***	***0.0470***	−0.3364	0.3132	**0.6514**	**0.0340**	***0.7936***	***0.0051***
**Flow cytometry (FCM)**								
#D+actNs	***−0.8033***	***0.0044***	**−0.6273**	**0.0440**	0.6052	0.0527	0.5367	0.0920
%D+actNs	***−0.7657***	***0.0084***	−0.6091	0.0519	0.5174	0.1061	0.4220	0.1961
%D+act[Ns+Ms]	***−0.8737***	***0.0009***	**−0.7545**	**0.0098**	0.4250	0.1928	0.5367	0.0920
%D+actMs	**−0.7298**	***0.0142***	***−0.7973***	***0.0048***	0.4491	0.1658	**0.7379**	**0.0120**
**Immunofluorescence Cytology**								
#D+actNETosing Ns	***−0.8880***	***0.001***	***−0.8090***	***0.0039***	0.5960	0.057	***0.7750***	***0.007***
Circularity index	**0.7140**	**0.0174**	**0.7091**	**0.0182**	−0.2264	0.5006	−0.5230	0.1020

n = 11 subjects. #, number (K/mL) whole blood; %, % positive among all neutrophils (Ns) or monocytes (Ms); D+, total (cell surface + intracellular) DEspR+ expression; #D+actNs, DEspR+CD11b+ neutrophil (K/mL); %D+actMs, %DEspR+CD11b+ monocytes among all monocytes; NLR, neutrophil to lymphocyte ratio = absolute neutrophil/absolute lymphocyte; #D+actNETosing Ns (K/mL): number of DEspR+CD11b+ NETosing Ns, calculated as follows: (#D+Ns x %NETosing Ns)/100.

**Clinical measures of severity:** ICU-free days at day 28 = [28 – number of ICU days] with NonSurvivors = −1 and Survivors > 28 ICU days = 0. S/F ratio, SpO2/FiO2 ratio as a measure of hypoxemia severity. t1-SOFA, sequential organ failure assessment (SOFA) score on day of flow cytometry analysis; t2-SOFA, SOFA score at end of ICU stay (day before ICU-discharge or ICU-death).

**Statistical analysis:** Spearman Rank Order Correlation coefficient *rho* (r_S_) effect size: strong r_s_ 0.6 – 0.79; very strong r_s_ 0.8 – 1.0 monotonic relation between paired data; (−r_S_) negative monotonic relation between paired data. Data points are peak values for subjects with multiple FCM analyses. Spearman Correlation Coefficient r_S_ > 0.758, alpha < 0.05, Power > 0.8 (*red italics*); r_S_ > 0.6, alpha < 0.05, power 0.7 to 0.8 (blue).
